# Guillain–Barré Syndrome and Variants Following COVID-19 Vaccination: Report of 13 Cases

**DOI:** 10.3389/fneur.2021.820723

**Published:** 2022-01-27

**Authors:** Jee-Eun Kim, Young Gi Min, Je-Young Shin, Young Nam Kwon, Jong Seok Bae, Jung-Joon Sung, Yoon-Ho Hong

**Affiliations:** ^1^Department of Neurology, Seoul Hospital, Ewha Womans University College of Medicine, Seoul, South Korea; ^2^Department of Neurology, Seoul National University Hospital, Seoul, South Korea; ^3^Department of Translational Medicine, Seoul National University College of Medicine, Seoul, South Korea; ^4^Department of Neurology, Kangdong Hanlym University Hospital, Seoul, South Korea; ^5^Department of Neurology, Seoul National University College of Medicine, Seoul National University Seoul Metropolitan Government Boramae Medical Center, Seoul, South Korea

**Keywords:** SARS-CoV-2, COVID-19, vaccination, COVID-19 vaccines, Guillain-Barré syndrome

## Abstract

**Background:**

Amidst growing concern about an increased risk of Guillain–Barré syndrome (GBS) following COVID-19 vaccination, clinical and electrodiagnostic features have not been fully characterized.

**Methods:**

We retrospectively reviewed medical records of the patients diagnosed with GBS and its variants following COVID-19 vaccination at four referral hospitals during the period of the mass vaccination program in South Korea (February to October 2021).

**Results:**

We identified 13 patients with GBS and variants post COVID-19 vaccination: AstraZeneca vaccine (Vaxzevria) in 8, and Pfizer-BioNTech vaccine (Comirnaty) in 5. The mean time interval from vaccination to symptom onset was 15.6 days (range 4–30 days). Electrodiagnostic classification was demyelinating in 7, axonal in 4 and normal in 2 cases. Clinical manifestations were diverse with varying severity: classical GBS in 8 cases, paraparetic variant in 3, Miller-Fisher syndrome in 1 and acute cervicobrachial weakness in 1. Four patients developed respiratory failure, and 2 of them showed treatment-related fluctuations.

**Conclusion:**

Our observations suggest that COVID-19 vaccines may be associated with GBS of distinctive clinical features characterized by severe quadriplegia, disproportionately frequent bilateral facial palsy or atypical incomplete variants. Continuous surveillance and further studies using robust study designs are warranted to fully assess the significance of the association.

## Introduction

Guillain–Barré syndrome (GBS) is the most common cause of acute flaccid paralysis with an incidence of 0.81 to 1.91 cases per 100,000 person-years (1). A respiratory or gastrointestinal infection precedes GBS onset in approximately two thirds of cases, which is thought to drive an aberrant autoimmune response against the peripheral nerves (2). While COVID-19 is recognized to trigger GBS (3), cases of GBS emerged in conjunction with COVID-19 vaccines, particularly those using the adenoviral vectors. Since then, the association of GBS with COVID-19 vaccines has been under close scrutiny. As of July 31, 2021, a total of 833 cases of GBS had been reported with the AstraZeneca vaccine (Vaxzevria) worldwide, while ~592 million doses had been given to people worldwide (4). In a recent COVID-19 vaccine safety update by the European Medicines Agency (EMA), a causal relationship between Vaxzevria and GBS was considered at least a reasonable possibility (4). Within the US Vaccine Adverse Event Reporting System (VAERS), 130 cases of presumptive GBS following receipt of the Ad26.COV2.S vaccine (Janssen/Johnson & Johnson) were reported from February 2021 to July 2021 (5). Despite a growing concern for an increased risk of GBS post-COVID-19 vaccination, the current literature is limited to several case reports and a study based on a passive reporting system that is subject to presumptive case definition and underreporting (4–9). Herein, we undertook a review of 13 cases consecutively encountered at four referral centers during the period of mass vaccination in South Korea (February to October 2021) to investigate clinical and electrodiagnostic patterns of GBS following COVID-19 vaccination.

## Materials and Methods

We retrospectively reviewed the medical records of the patients with GBS and variants following COVID-19 vaccination at four referral hospitals (Seoul National University Hospital, Seoul Metropolitan Boramae Medical Center, Ewha Womans University Hospital and Hanlym University Hospital) during the period of the mass vaccination program in South Korea (February to October 2021). GBS was diagnosed according to the established criteria (10). The time interval from vaccination and GBS onset should be <42 days. We thoroughly investigated the history of preceding infection, and excluded four patients with antecedant gastrointestinal infection. There was none with a history of preceding respiratory infection. Electrodiagnostic classification was based on the criteria by Uncini et al. (11). Skin biopsy was performed to analyze the pathology of dermal myelinated fibers according to the in-house protocol. Briefly, we performed a 4 mm-sized punch biopsy 10 cm above the left lateral malleolus. The sample was washed with phosphate-buffered saline (PBS) and fixed in Zamboni's solution for 24 h at 4°C. It was then embedded in 20% sucrose PBS, and cut in 50 μm thick sections using a cryotome. Free-floating sections were incubated for 24 h with two sets of primary antibodies to axon (protein gene product 9.5; PGP9.5), myelin (myelin basic protein; MBP), nodes of Ranvier (voltage gated sodium channel; Nav), or myelin (MBP), paranode (contactin-associated protein; Caspr), and nodes of Ranvier (Nav). Alexa Flour dyes were used as secondary antibodies to visualize the markers. Using a confocal microscope (Zeiss, Oberkochen, Germany), Z-stack series images of the sections were acquired at 1-μm increment. Antibody sources and dilutions are shown in the Supplementary Table 1.

## Results

We identified 13 patients with GBS post COVID-19 vaccination (4 males, 9 females): 8 patients received the AstraZeneca vaccine (Vaxzevria) and 5 received the Pfizer-BioNTech vaccine (Comirnaty). The clinical features of these 13 patients are summarized in Table 1 and Supplementary Table 2. Mean age was 56.4 years (range 18–84 years). The mean time interval from vaccination to GBS onset was 15.6 days (range 4–30 days). All patients except one developed GBS after receiving the first dose of a COVID-19 vaccine. A cerebrospinal fluid (CSF) analysis was conducted on mean 10.9 days (range 5–40) after symptom onset in 12 patients. Albuminocytologic dissociation was detected in 9 of 12 patients. Nerve conduction studies (NCS) were performed in all patients (serial studies in 10 patients). Applying the criteria by Uncini et al. the electrodiagnostic classification was demyelinating in 5 patients, axonal in 3 patients and normal in 2 patients (one with Miller-Fisher syndrome and the other with mild paraparetic variant). Three patients (Case 7, 9, 12) who did not receive a follow-up NCS were classified as “undetermined.” Anti-ganglioside antibodies were positive in 2 patients (anti-GM1 IgM antibody in one axonal GBS, and anti-GQ1b IgG antibody in one Miller-Fisher syndrome). Overall, the clinical manifestations were diverse with varying severity. Eight patients showed the classical GBS phenotype, while the other 5 patients showed distinct clinical features of GBS variants (3 with paraparetic variant, 1 with Miller-Fisher syndrome and 1 with acute cervicobrachial weakness). Four patients (30.8%) developed respiratory failure and required mechanical ventilation in an intensive care unit. Two of these severe GBS patients showed treatment-related fluctuations (TRF). Cranial nerve involvement was common, and facial paresis occurred in 4 patients (one patient with bifacial diplegia). One patient (Case 11) died of aspiration pneumonia. Four patients (Case 1, 2, 10, 13) were still unable to walk at 2 months from disease onset. Nasopharyngeal swabs for SARS-CoV-2 were performed in all patients by real-time polymerase chain reaction tests, and the results were all negative. Four specific cases worth mentioning are presented below.

**Table 1:** Clinical features of 13 patients with Guillain–Barré syndrome and variants post COVID-19 vaccination.

**Case**	**1**	**2**	**3**	**4**	**5**	**6**	**7**	**8**	**9**	**10**	**11**	**12**	**13**
Age	62	73	48	32	38	70	72	84	62	43	18	73	58
Gender	M	F	F	F	M	F	M	F	F	M	F	F	F
Time from vaccination to symptom onset (days)	11	29	10	11	29	8	30	8	27	11	12	13	4
Vaccine	ChAdOx1-S/nCoV-19 (1st)	ChAdOx1-S/nCoV-19 (1st)	ChAdOx1-S/nCoV-19 (1st)	BNT162b2 (1st)	ChAdOx1-S/nCoV-19 (1st)	ChAdOx1-S/nCoV-19 (1st)	ChAdOx1-S/nCoV-19 (1st)	ChAdOx1-S/nCoV-19 (1st)	ChAdOx1-S/nCoV-19 (2nd)	BNT162b2 (1st)	BNT162b2 (1st)	BNT162b2 (1st)	BNT162b2 (1st)
Clinical phenotype	Classic GBS	Classic GBS	Paraparetic GBS	Acute cervicobrachial weakness	Classic GBS	Classic GBS	Classic GBS	Classic MFS	Classic GBS	Classic GBS	Paraparetic GBS	Paraparetic GBS	Classic GBS
Antiganglioside antibody^*^	Negative	Negative	Negative	Negative	Negative	GM1 IgM(+)	NA	GQ1b IgG(+)	Negative	Negative	Negative	Negative	Negative
EDX classification^†^	AMAN	AIDP	AIDP	AIDP	AMAN	AMAN	Undetermined	Normal	Undetermined	AIDP	Normal	Undetermined	AIDP
TRF (days from relapse after last day of 1st IVIG)	Yes (7)	Yes (7)	No	No	No	No	No	No	No	No	No	No	No
Onset to nadir (days)	10, 22^‡^	7, NA^‡^	15	12	1	11	10	5	11	21	9	9	4
**Severity at nadir**													
Hughes score	5	4	2	1	2	3	2	1	4	4	2	3	5
MRC sum score	14	28	57	51	56	54	52	60	52	26	56	46	18
Length of hospital stay	139	42	9	6	6	20	2	17	6	20	3	10	44
Treatment	First and second IVIG	IVIG	Steroid	IVIG	No	IVIG	No	No	IVIG	IVIG	No	IVIG	IVIG followed by plasmapheresis
Onset to improvement (days)	16, 30^∫^	15, 26^∫^	18	17	16	18	25	30	43	23	13	15	6
**Prognosis at 2 months**													
Hughes score	4	3	1	1	0	2	1	1	1	3	1	6	3
MRC sum score	26	44	60	60	60	58	60	60	59	48	58	0	35

*AIDP, acute inflammatory demyelinating polyradiculoneuropathy; AMAN, acute motor axonal neuropathy; EDX, electrodiagnostic study; F, female; IVIG, intravenous immunoglobulin G; GBS, Guillain–Barré syndrome; M, male; MRC, medical research council; NA, not available; TRF, treatment-related fluctuations*.

* *The anti-GM1, GD1b, GQ1b IgG, and IgM panels were checked with ELISA for anti-ganglioside antibody assays*.

† *Electrodiagnostic classification was determined by serial nerve conduction studies according to Uncini's criteria. ^11^Three patients who did not receive a follow-up nerve conduction study were classified as “undetermined”*.

‡ *Onset to nadir before and after treatment-related fluctuation, respectively*.

∫ *Time interval from onset to improvement following the first and second IVIG infusions, respectively*.

**Case 1**. A 62-year-old man developed acute onset of tingling and weakness in his hands and feet 10 days after the first dose of Vaxzevria, which progressed proximally with severe back pain. Shortly thereafter, he complained of altered taste and paresthesia in the tongue and perioral area and developed hoarseness and dysphagia. Over the next 10 days after admission, the weakness progressed rapidly to areflexic quadriplegia and bifacial diplegia and ultimately respiratory failure. A nerve conduction study was performed 2 months later, and the result was consistent with axonal GBS by the Uncini criteria (11). An additional course of intravenous immunoglobulin (IVIG) infusions was administered due to worsening of the weakness after initial transient improvement (TRF). He was discharged in a bedridden state after 4 months of hospitalized care.

**Figure 1:**
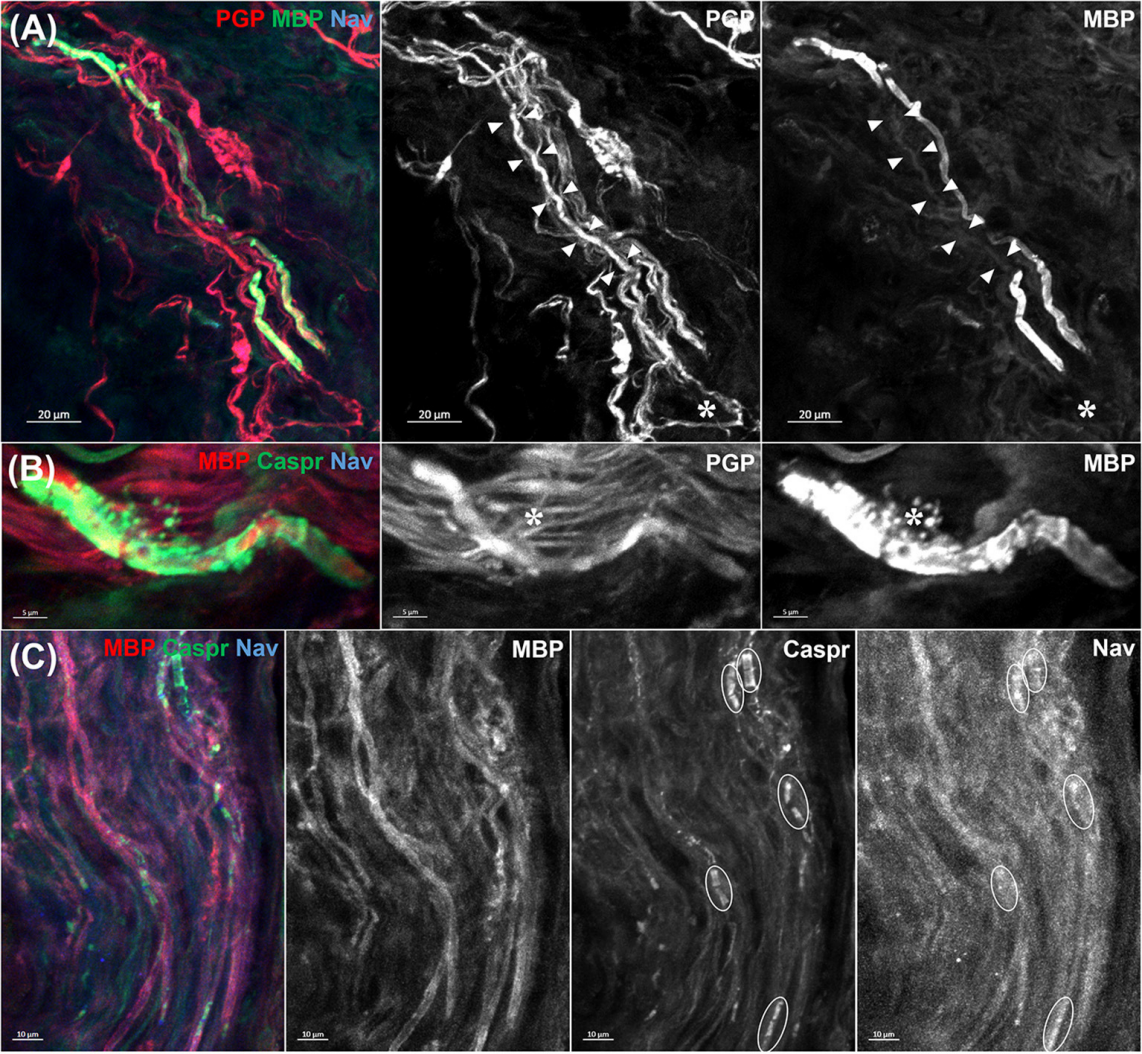
Morphology of dermal myelinated fibers in patient 2. A skin biopsy was performed 10 cm above the left lateral malleolus. Primary antibodies to axon (protein gene product 9.5; PGP9.5), myelin (myelin basic protein; MBP), node of Ranvier (voltage gated sodium channel; Nav), paranodes (contactin-associated protein; Caspr) and secondary antibodies (Alexa Fluor dyes) were used. **(A)** A denuded axon flanked by myelin sheath (arrowheads) indicates a segmental demyelination. Another demyelinated segment is seen at the bottom of picture (asterisk). **(B)** Ongoing myelin breakdown with preserved axonal integrity (asterisk) was also noted. **(C)** In contrast, nodes of Ranvier and paranodes (circles) showed no abnormal dispersion or loss. The above pathological findings favored the diagnosis of acute inflammatory demyelinating polyradiculoneuropathy rather than nodal/paranodal Guillain–Barré syndrome.

**Case 2**. A 73-year-old woman presented with distal paresthesia and weakness in four limbs after 29 days after the first dose of Vaxzevria. Her weakness rapidly progressed in an ascending pattern, and ultimately led to respiratory failure. The results of the nerve conduction study were consistent with acute inflammatory demyelinating polyradiculoneuropathy (AIDP). A skin biopsy performed 8 days after symptom onset also revealed the pathology of segmental demyelination in dermal myelinated nerve fibers without nodal and paranodal structure destruction (Figure 1).

**Case 3**. A 48-year-old woman experienced severe pain as if a band was wrapped around her chest, 14 days after the first dose of Vaxzevria. Six days later, she developed weakness in both lower extremities (MRC 4/4-) and electrical shock-like pain radiating from both posterior hips to the soles. She also noticed facial paresis on the left side. The result of serial nerve conduction study was consistent with AIDP. IVIG treatment was withheld because her weakness was mild, and oral prednisolone was administered for facial palsy.

**Case 4**. A 32 year-old woman felt shoulder pain with weakness in her left arm 11 days after receiving Comirnaty. Initially, she felt left elbow flexion weakness which progressed proximally limiting shoulder movements over the next 9 days. She also felt her right arm was weaker than usual. A neurological examination showed asymmetric arm weakness (proximal MRC 4/2, distal MRC 5/5) with areflexia. Oropharyngeal or leg weakness were not detected. A nerve conduction study showed the features of primary demyelinating polyneuropathy in four limbs. Albuminocytologic dissociation was not detected in cerebrospinal fluid study that performed 9 days after symptom onset. She was diagnosed as acute cervicobrachial weakness and treated with IVIG. Five days after starting IVIG treatment, the strength in the left arm started to slowly improve.

## Discussion

As of October 31, 2021, about 41 million people (79.4% of the population) had received their first doses of the COVID-19 vaccines (11 million Vaxzevria; 22 million Comirnaty; 1.5 million Ad26.COV2.S; 6.5 million Moderna COVID-19 vaccines) in South Korea (12). The time interval between vaccination and GBS onset ranged from 4 to 30 days in our patients, which is in line with previous case reports and coincides with the expected time course of the immune response to COVID-19 vaccines (6–9). GBS was more frequently reported in adenovirus-vector vaccines, but there are also several case reports GBS with mRNA vaccines (6–9). In this study, we identified 13 patients who developed GBS and variants within 42 days of COVID-19 vaccination during the study period (8 patients receiving Vaxzevria and 5 patients receiving Comirnaty vaccinations). All patients except one developed GBS after receipt of the first dose of the COVID-19 vaccines.

Our observations are in line with previous small case series and reports, supporting that 1) COVID-19 vaccines, particularly the first dose of adenoviral vector-based ones, may be associated with GBS, and 2) GBS post COVID-19 vaccination may have distinctive clinical features. According to a recently published large population-based UK study, which is based on English National Immunization (NIMS) Database of COVID-19 vaccination, the rate of GBS following Vaxzevria vaccination was higher than the background rates within 28 days of the first dose, with the risk ~38 excess cases per 10 million exposed (13).

Maramattom et al. reported that all seven patients (Vaxzevria vaccine) progressed to areflexic quadriplegia and bilateral facial paresis and six of them required mechanical ventilation for respiratory failure (6). Allen et al. and Bonifacio et al. reported 4 and 5 cases, respectively, of the bifacial weakness with paresthesias (BFP) variant (Vaxzevria vaccine) (7, 14). Although we did not observe any case with BFP variant, severe GBS and atypical incomplete variants were relatively common in our experience. Paraparetic variant, reportedly occurs in 5–10% of GBS cases, was observed in 3 patients (23.1%), while acute cervicobrachial weakness and Miller-Fisher syndrome were also identified. Intriguingly, treatment-related fluctuations (TRF) were observed at a rather high rate (2 of 13 patients, 15.4%) compared to the previously known figures (5.3–16%) (15–17). Larger studies are needed to determine whether TRF are more common in the COVID-19 vaccination-associated GBS.

Electrodiagnostic classification of GBS following COVID-19 vaccination was reported to be demyelinating in most cases (6, 7, 18). However, our experience was rather different with three cases of the primary axonal subtype (all with Vaxzevria). Differences in the genetic background, timing of serial nerve conduction studies and electrodiagnostic criteria may account for the discrepancy. The immunopathogenesis of GBS post COVID-19 vaccination is unknown, but may involve a cross-reactivity to the axonal or myelin components of the peripheral nerves (19). Given the higher risk of GBS following the adenovirus-vector vaccines, it is more likely that the immune response to the adenoviral vectors rather than the SARS-CoV-2 spike protein may be involved in the pathogenesis of GBS post COVID-19 vaccination.

This study is a retrospective case series, and because of this limitation we cannot establish robust causal relationships between GBS and COVID-19 vaccination. Other limitations include the lack of comprehensive serological tests for antecedent infections, although we excluded symptomatic respiratory or gastrointestinal infections by thorough investigation of medical history. Overall, our observations corroborate the findings of previous case reports, suggesting that COVID-19 vaccines may be associated with GBS of distinctive clinical features characterized by severe quadriplegia, disproportionately frequent bilateral facial palsy or atypical incomplete variants. Knowledge of this rare potential side effect is important for early diagnosis and proper treatment. Continuous surveillance and further studies using robust study designs are warranted to fully assess the significance of the association.

## Data Availability Statement

The raw data supporting the conclusions of this article will be made available by the authors, without undue reservation.

## Ethics Statement

The studies involving human participants were reviewed and approved by the Seoul National University Hospital, Seoul Metropolitan Boramae Medical Center, Ewha Womans University Seoul Hospital, and Kangdong Sacred Heart Hospital review board (IRB# SNUH & BRMH-2011-067-1271; EUMC-2021-11-006; KSHH-2021-11-003, respectively). The patients/participants provided their written informed consent to participate in this study.

## Author Contributions

Y-HH had full access to all of the data in the study and takes responsibility for the integrity of the data and the accuracy of the data analysis. Concept and design: J-EK, YM, and Y-HH. Acquisition, analysis, or interpretation of data: J-EK, YM, J-YS, YK, and JB. Drafting of the manuscript and statistical analysis: J-EK and YM. Critical revision of the manuscript for important intellectual content: J-YS, YK, J-JS, JB, and Y-HH. Administrative, technical, or material support: J-EK, YM, J-JS, YK, and JB. Supervision: JB, J-JS, and Y-HH. All authors contributed to the article and approved the submitted version.

## Conflict of Interest

The authors declare that the research was conducted in the absence of any commercial or financial relationships that could be construed as a potential conflict of interest.

## Publisher's Note

All claims expressed in this article are solely those of the authors and do not necessarily represent those of their affiliated organizations, or those of the publisher, the editors and the reviewers. Any product that may be evaluated in this article, or claim that may be made by its manufacturer, is not guaranteed or endorsed by the publisher.

## Abbreviations

AIDP: acute inflammatory demyelinating polyradiculoneuropathy
BFP: bifacial weakness with paresthesias
IVIG: intravenous immunoglobulin G
GBS: Guillain–Barré syndrome
MRC: medical research council
TRF: treatment-related fluctuations.
