# Supplementary material for: Guillain–Barré Syndrome and Variants Following COVID-19 Vaccination: Report of 13 Cases
Source: Front Neurol. 2022 Jan 27;12:820723. doi: 10.3389/fneur.2021.820723 (PMC8833101; doi:10.3389/fneur.2021.820723)
Supplement: Supplementary file 1 [file Table_1.DOCX]

Supplementary Material

# Supplementary Table 1. Antibody sources and dilutions used for skin biopsy in case 2 patient.

| **Primary antibody (Abbreviation)** | **Manufacturer** | **Dilution** | **Secondary antibody**  (combination 1) | **Secondary antibody**  **(**combination 2) | **Manufacturer** | **Dilution** |
| --- | --- | --- | --- | --- | --- | --- |
| Guinea pig protein gene product 9.5 (PGP9.5) | GeneTex, Irvin, USA | 1:500 | Alexa 568 |  | Invitrogen, Madison, USA | 1:300 |
| Rat myelin basic protein (MBP) | GeneTex, Irvin, USA | 1:600 | Alex 488 | Alexa 568 |  |  |
| Mouse voltage gated sodium channel (Nav) | Sigma-Aldrich, St.Louis, USA | 1:100 | Alexa 647 | Alexa 647 |  |  |
| Rabbit contactin-associated protein (Caspr) | Abcam, Cambridge. UK | 1:100 |  | Alexa 488 |  |  |

**Supplementary Table 2. Clinical features of 13 patients with Guillain–Barré syndrome and variants post COVID-19 vaccination.**

| Case | 1 | 2 | 3 | 4 | 5 | 6 | 7 | 8 | 9 | 10 | 11 | 12 | 13 |
| --- | --- | --- | --- | --- | --- | --- | --- | --- | --- | --- | --- | --- | --- |
| Clinical features | | | | | | | | | | | | | |
| Limb weakness | quadriparesis | quadriparesis | lower leg weakness, MRC grade 4/4- | bilateral upper extremity weakness (proximal MRC grade 4/2, distal 5/5) | distal hand, leg weakness | distal hand, leg weakness | quadriparesis with dysarthria | no | both hands weakness (MRC grade 4/4), right leg weakness (MRC grade: knee extension 3, ankle dorsiflexion 1) | quadriparesis | distal leg symmetric weakness | lower leg weakness, MRC grade 2/3 | quadriparesis |
| Limb sensory changes | distal hands & feet tingling, ascending | distal hands & feet tingling, ascending | both posterior hip - sole tingling, trunk pain | no | no | no | pain | no | severe loss of proprioception on both legs | decreased pain and vibration sensation over entire legs | no | decreased pain and vibration sensation over entire legs | distal hands & feet tingling, ascending |
| Cranial neuropathy | bifacial diplegia, hoarsenes, dysphagia, perioral/  tongue numbness | no | facial diplegia | no | no | no | no | complete opthalmoplegia with bilateral ptosis | no | facial diplegia, trigeminal motor | no | no | facial diplegia, dysphagia |
| Radicular pain | yes | yes | yes | yes | no | no | yes | no | yes | yes | no | no | no |
| Respiratory failure | yes | yes | no | no | no | no | no | no | no | yes | no | no | yes |
| Dysautonomia | yes | yes | no | no | no | no | no | no | yes | no | no | no | no |
| SIADH | yes | yes | no | no | no | no | yes | yes | no | no | no | no | no |
| Laboratory findings | | | | | | | | | | | | | |
| Time of CSF study from onset (days) | 5 | 7 | 9 | 9 | 5 | 12 | NA | 11 | 40 | 6 | 14 | 10 | 3 |
| CSF protein (mg/L) | 90 | 89 | 111 | 42 | 33 | 95.6 | NA | 58 | 31 | 151 | 29 | 126 | 48 |
| CSF white cells (/mm^3^) | 0 | 2 | 0 | 0 | 1 | 1 | NA | 0 | 0 | 2 | 0 | NA | 0 |
| Spine MRI | intense enhancement in lumbar and cauda equina | mild cauda equina thickening and enhancement | normal | cauda equina thickening and enhancement | cauda equina thickening and enhancement | cauda equina thickening and enhancement | NA | NA | normal | NA | NA | normal | normal |
| Serial EDX | | | | | | | | | | | | | |
| 1st EDX from onset (days) | 65 | 7 | 10 | 10 | 5 | 11 | 30 | 12 | 41 | 6 | 6 | 13 | 5 |
| 2nd EDX from onset (days) | 85 | 14 | 17 | 22 | 12 | 18 | NA | 23 | NA | 10 | 16 | NA | 16 |
| 3rd EDX from onset (days) | 125 | 19 | 39 | NA | 52 | 45 | NA | NA | NA | 24 | NA | NA | 23 |

CSF, cerebrospinal fluid; EDX, electrodiagnostic study; MRC, medical research council; NA, not available; SIADH, syndrome of inappropriate antidiuretic hormone.
